# Facilitators to Accessibility of HIV/AIDS-Related Health Services among Transgender Women Living with HIV in Yogyakarta, Indonesia

**DOI:** 10.1155/2019/6045726

**Published:** 2019-07-01

**Authors:** Nelsensius Klau Fauk, Maria Silvia Merry, Theodorus Asa Siri, Fabiola Tazrina Tazir, Mitra Andhini Sigilipoe, Kristin Oktanita Tarigan, Lillian Mwanri

**Affiliations:** ^1^College of Medicine and Public Health, Flinders University, GPO Box 2100, Adelaide 5001, South Australia, Australia; ^2^Institute of Resource Governance and Social Change, Jl. R. W. Monginsidi II, Kupang, Nusa Tenggara Timur, 85221, Indonesia; ^3^Medicine Faculty, Duta Wacana Christian University, Jl. Doktor Wahidin SudiroHusodo, Yogyakarta, 55224, Indonesia; ^4^Saint Peter Pastoral Institute of the Diocese of Atambua, Jl. Eltari, Kefamenanu, Nusa Tenggara Timur, 85613, Indonesia; ^5^National Population and Family Planning Board, Jl. Permata No. 1, Halim Perdana Kusuma, Jakarta Timur, 13650, Indonesia; ^6^BPS-Statistics Indonesia, Jl. Sutomo No. 6-8, Sawah Besar, Jakarta Pusat, 10710, Indonesia

## Abstract

The study aimed to explore facilitators or enabling factors that enhance accessibility (defined as the opportunity to be able to use) to HIV/AIDS-related health services among HIV positive transgender women, also known as* Waria* in Yogyakarta, Indonesia. A qualitative study employing one-on-one in-depth interviews was conducted from December 2017 to February 2018. Participants were HIV positive* Waria* recruited using purposive and snowball sampling techniques. Data were analysed using the framework analysis for qualitative research. The findings showed that participants' knowledge of HIV/AIDS and the availability of HIV/AIDS-related health services were enablers to the services accessibility. Emotional support from fellow* Waria* displayed in various ways, such as kind and caring attention, attentive listening, and encouraging words, was an important social support that played a role in supporting* Waria's* accessibility to the services. HIV/AIDS-related health service information shared personally or jointly by fellow* Waria* and instrumental support including helping each other to collect antiretroviral (ARV) from hospitals or community health centres, contacting ambulance in emergency situations, accompanying each other to health service facilities, and helping those without the health insurance to receive free health services were also the social support enabling accessibility to the services among the study participants. Appraisal support such as providing constructive feedback and affirmation was another enabling factor to* Waria*'s accessibility to the services. The findings indicate the needs to broadly disseminate information and educate* Waria* populations and their significant others about HIV/AIDS and related health services to raise their awareness of HIV/AIDS and acceptance of HIV/AIDS positive individuals. Educating and broadly disseminating this information in other settings in the country will also increase accessibility to the HIV/AIDS services among* Waria*, their families, and communities addressing the currently existing inequities in health. The findings also reinforce the importance of the establishment of* Waria* peer-support groups within* Waria* communities and the involvement of* Waria* in HIV/AIDS activities and programs, which may increase their awareness of HIV/AIDS, and accessibility to HIV/AIDS-related health services.

## 1. Introduction

The 2017 UNAIDS report shows that, between 2010 and 2016, there was a global reduction of, respectively, 32% AIDS-related deaths and 16% of HIV infections attributed to a scale-up access to HIV/AIDS-related services especially antiretroviral therapy (ART) coverage [1, 2]. In Asia and the Pacific region, a reduction of nearly one-third of AIDS-related deaths during the same period was also reported to be a result of the wider availability of ART [1]. However, Indonesia is one of the few countries in the region reported to have experienced a significant increase in AIDS-related deaths (68%) and HIV cases (316%) during the same period [1]. The increase in HIV infection cases has been noted to be higher in ‘HIV high-risk groups' including sex workers, clients of sex workers, and men who have sex with men, transgender persons (*Waria*) and those whose HIV/AIDS-related service accessibility (voluntary counselling and testing (VCT)) and ART [3–6] is difficult.

*Waria* (a combination of two Indonesian words:* WAnita* (woman) and* pRIA* (man)) are men who adorn themselves to appear as women or male individuals who generally dress and act in a normatively feminine manner [7].* Waria* often self-describe as having the body of a man but the soul of a woman [8]. Some authors have also used the term* Waria *to refer to “male-to-female transgenders” [9–12]. Generally,* Waria *are viewed as sexual deviants, people who are contaminated, and are often rejected in most cultures and societies in Indonesia. As a result, it is difficult for them to openly talk about their health status and to seek healthcare services [8, 13, 14]. Because of the stigma associated with being* Waria*, these populations live with vulnerability and are known to be at high risk for HIV infection in Indonesia. It is estimated that two million* Waria* live in Indonesia and 24.8% of them have been reported to live with HIV infections [1, 15]. Compared to the general population with the HIV prevalence of 0.15%, this is a significant proportion of a group of people living with HIV. It is thus reasonable to argue that* Waria* may face vulnerability or disadvantages similar to other known vulnerable populations described above.

Globally, availability of health services such as HIV counselling and testing and ART has been as a significant enabler to HIV/AIDS-related service accessibility [16]. Moreover, HIV/AIDS education and knowledge about HIV/AIDS have also been reported as facilitators of HIV/AIDS-related health service accessibility among different populations, such as students, men who have sex with men, sex workers, and transgender persons [17–20]. Social support from friends, sex partners, families, and communities has also been demonstrated to play an important role in facilitating HIV/AIDS-related service accessibility [21–24].

Evidence exists illustrating the lack of HIV/AIDS-related health services among* Waria* populations in many parts of Indonesia [25, 26] except in Yogyakarta, where these services are reported to be available and have easy accessibility [27, 28]. Promoting accessibility to HIV/AIDS-related health services to* Waria* would be one among important HIV prevention strategies to reduce HIV infections in Indonesia. To improve our knowledge about what makes these services to be effectively useful within populations, this study aimed to identify facilitators to HIV/AIDS-related health service accessibility among* Waria* living with HIV in Yogyakarta. Overall goal was to gain an insight about strategies and interventions that promote HIV/AIDS service accessibility among* Waria* populations in Indonesia.

## 2. Methodology

Consolidated criteria for reporting qualitative studies (COREQ) checklist for explicit and comprehensive reporting of qualitative studies especially interviews and focus groups were employed to guide the report of the methodological section of this study [29].

### 2.1. Theoretical Framework

This study was guided by theoretical assumptions, including the social support framework and the Health Belief Model (HBM). Social support is an important function for sustained relationships among individuals [30, 31]. It is the functional content of relationships and can be categorised in four major types of supportive behaviours of acts, including emotional, informational, instrumental, and appraisal support [30, 32, 33].* Emotional support* refers to the provision of empathy, love, trust, and caring for people in need. In the context of this study, the* emotional support* involves kind acts of attention, caring, listening, and encouraging words from family members and friends.* Instrumental support* refers to the provision of tangible aid and services that directly assist other people in need.* Informational support* involves the provision of advice, suggestions, and information that a person can use to address problems.* Appraisal support* involves the provision of information or constructive feedback and affirmation useful for self-evaluation purposes [30, 32, 33].

Furthermore, the HBM suggests that individual's health behaviour is determined by several key perceptions or beliefs about the condition or disease including perceived susceptibility, perceived severity, perceived benefits, perceived barriers, cues to action, and self-efficacy [34, 35]. Perceived susceptibility refers to a person's perception of the possibility to contracting a disease (in the context of current study participants: HIV/AIDS) if he or she does not undertake a recommended action, such as using condoms to prevent HIV transmission. Perceived severity refers to an individual belief of the seriousness of a given disease and its impact on his or her health. The greater the belief, the more likely a person is to take the recommended behaviours or actions and vice versa. Perceived benefits are about an individual's perception of advantages of undertaking an action or performing a behaviour. Perceived barriers are related to personal perception of factors that may hinder a person from performing a behaviour. Factors such as costs of undertaking required actions could be barriers, for example, to accessing antiretroviral treatment for people living with HIV. Cues to action may refer to motivation for action or behavioural changes. Self-efficacy refers to an individual belief in his or her ability to successfully perform a recommended action or behaviour. These perceptions or beliefs may also be influenced by other aspects, including demographic, sociopsychological, and structural factors [34, 35]. For instance, the lack of availability of HIV/AIDS-related health facilities and services can be one of the structural factors that influence an individual's perception to accessibility of the services.

### 2.2. Study Setting

Yogyakarta is a province in Indonesia located in Jave Island, with a population of 636,660 at the density of 13,340 people/km^2^. The province comprises four districts, one municipality, 78 subdistricts, and 438 villages and has 74 hospitals comprising 60 private hospitals and 14 government hospitals and 121 community health centres [36, 37]. Of the hospitals and community health centres, four hospitals and 10 community health centres provide HIV/AIDS-related health services [38]. The services provided include health information on HIV/AIDS and the related services, HIV counselling and testing, CD4 test, viral load test, and ART. HIV/AIDS-related health information services are provided through workshops for people living with HIV/AIDS and regular focus groups discussion with the* Waria* community.

### 2.3. Study Design, Recruitment, and Data Collection

This inquiry was conducted from December 2017 to February 2018 in Yogyakarta, Indonesia, using a qualitative study design. The use of a qualitative design was useful, providing the researchers with opportunities to have direct interaction with the study participants and observe the situation and the setting where they lived, worked, and interacted [39].

The study participants were* Waria* recruited using a purposive, followed by the snowball sampling, technique. Initially, a* Waria* coordinator of HIV service in the city of Yogyakarta was purposively approached by the first two authors (NK and MSM). The coordinator was also the head of a nongovernmental organisation (NGO) providing support for* Waria* populations and people living with HIV/AIDS. The support included providing information of HIV/AIDS and HIV/AIDS-related services, procedures how to use HIV/AIDS-related services, and linking people living with HIV/AIDS to available healthcare services from facilities, such as hospitals and community health centres. The coordinator was asked to distribute information sheet containing a brief about the study and details of the first two authors. The study information sheet was put on the information board at the office of this NGO to be taken away by potential participants who utilised the service of the NGO and on the information board at a* Waria's* shelter belonging to the NGO. Six initial potential participants (first wave) contacted the researchers in the next five days and stated their willingness to participate in this study. The six participants were asked to distribute the study information sheet to other people they knew. The same approach was used to recruit subsequent potential participants. Sixteen and ten other potential participants contacted the researchers in the second and third wave, respectively. Overall, a total of 32 potential participants indicated their willingness to participate. However, three potential participants withdrew their participation before being interviewed due to personal reasons. Finally, 29 people provided informed consent and willingness to participate and were interviewed and audio recorded. Participants were included when they met the following criteria: (i) HIV positive* Waria, *(ii) 18^+^ years old, and (iii) accessing the HIV/AIDS-related health services. The study included only the participants who have accessed HIV-related health services due to the existing evidence showing that the services in the study setting are available and accessible to* Waria *and other populations with HIV compared to many other parts of Indonesia [25–28]. Their insights can improve our understanding of factors supportive of HIV-related health service accessibility and help in the development of strategies and interventions that promote the utilisation of the services among HIV positive* Waria* and other populations in other parts of Indonesia and globally. Recruitment of study participants was stopped as the last few participants provided information or responses which were similar to those of previous participants, which indicated that data saturation had been reached. There were no prior relationships between any of the participants and researchers.

One-on-one interviews were held at the time and place suggested by each individual. Interviews were conducted by two senior researchers (NKF and MSM) who are trained in qualitative methods and experienced in HIV-related research, and have educational background in medicine and public health. Interviews focused on several key areas, including knowledge of HIV/AIDS, information of the availability of HIV/AIDS-related health services, how they obtained information of the services, how they accessed the services, and what enablers to the service accessibility were, including the role that* Waria* friends, families, and communities played in helping them in the service accessibility. No repeat interviews were conducted and none of the participants required to comment or correct interview transcript.

### 2.4. Data Analysis

The recorded interviews were transcribed verbatim and translated into English by the first two authors who are fluent in both Bahasa and English. To maintain the quality and validity of the data, data were cross-checked and comparisons were made between the first two authors during the transcription and translation process. The transcriptions and translations were also checked for accuracy and meaning by other authors. Guided by Braun and Clarke thematic framework analysis [40], the data analysis involved six phases. The first phase was* familiarisation* with the data or transcripts performed through repeated readings, note taking, marking ideas, and giving comments to search for meanings, patterns, and ideas. The second phase was* generation of initial codes* to the extracts of data from individual transcripts by writing notes on the texts being analysed. The third phase involved* searching for themes* where codes referring to the same theme or subtheme were grouped together, and all the relevant coded data extracts within the identified themes or subthemes were collated. The fourth phase involved a* review of themes and subthemes* by reading all the collated extracts for each theme to see whether or not they appeared to form a coherent pattern. This was to ensure that the themes were really themes supported by sufficient data and to see whether the themes could collapse into each other (e.g., two apparently separate themes might form one theme) or could be broken down to separate themes. For instance, knowledge of HIV/AIDS and sources of information about the HIV/AIDS-related service themes were combined under the theme knowledge of HIV/AIDS and HIV/AIDS-related health services for* Waria*. The fifth phase* defined and named the themes*. At this phase, the essences of what each theme was about (as well as themes overall) were identified and what aspect of the data each theme captured was determined. This allowed the researhers to ensure that each theme was not too diverse and complex. This was performed by going back to collated data extracts for each theme and organising them into a coherent and internally consistent account, with accompanying narrative. This step resulted in the collection of four final themes and four subthemes as presented in the result section. The sixth phase involved* producing the report*.

### 2.5. Ethical Consideration

Prior to the interviews, participants were informed about the purpose of the study and that ethics approval for this study was obtained from Medicine Research Ethics Committee, Duta Wacana Christian University, Indonesia (ref: 558/C.16/FK/2017). They were advised that their participation in this study was voluntary and that they could withdraw their participation during the interview if they felt uncomfortable about the topics being asked without any consequences. Before commencing each interview, participants were informed about the length of time that the interview would take (~ 45 to 90 minutes) and that this would be recorded using a tape-recorder and notes would be taken by the interviewer during the interview process. They were also assured that the collected information would be treated confidentially and anonymously by assigning each participant with a unique Study Identification Number (e.g., R1, R2,  etc.). This was to ensure that the information provided will not be linked back to each individual in the future. The participants signed and returned a written informed consent at the interview day.

## 3. Findings

### 3.1. Profile of the Respondents

Participants mean age was 43.99 (32-57 ± 5.9) years, with median of 44 years. Participants were originally from eight different provinces in Indonesia, including the Special Region of Yogyakarta (38%), Central Java (21%), West Java (14%), North Sumatera (7%), South Sumatera (7%), East Java (7%), Riau Islands (3%), and Bengkulu (3%). The education backgrounds varied with the composition of participants divided as follows: Senior High School and Junior High School graduates, respectively, being 31%; Elementary School graduates comprising 21%; and Elementary School dropouts being 17%. All the participants indicated to have sex work as one of the sources of income, but they also had part-time jobs, including makeup stylists, barbers at beauty salons, housekeepers, poultry and coconut sellers, batik dress sellers, and food venders. Several participants were volunteers at two NGOs providing supports for* Waria* populations and people living with HIV/AIDS. All the participants were living with HIV, with some reporting to having other sexually transmitted infections, such as syphilis, gonorrhoea, and genital warts. A few of participants mentioned to have been diagnosed with tuberculosis. For the purpose of this paper, four major themes with subsequent subthemes are presented below.

### 3.2. Knowledge about HIV/AIDS and HIV/AIDS-Related Health Services for* Waria*

Knowledge of HIV/AIDS and availability of HIV/AIDS-related health services seemed to be a facilitator to the service accessibility of* Waria*. It appeared that all the participants had the basic knowledge of HIV/AIDS. They also knew about the HIV/AIDS-related health services that were available for them. This knowledge was a result of receiving information about the disease and what services they were entitled to:

“I did not hear of HIV/AIDS health services when I was in my place of origin. I started knowing about HIV/AIDS and the services after I moved here. Knowledge or information I got helped me to make decision to undergo HIV counselling and testing” (R7: 45 years old).

“.... I decided to use the services after I received information about HIV/AIDS and the possible services I can access. I would have done this long time ago if I had this information before” (R10: 53 years old).

“I know about HIV/AIDS services here and use them regularly. I use ARV, check my CD4 level, and attend counselling. I am lucky to be exposed to information related to HIV/AIDS and HIV/AIDS services” (R11: 33 years old).

*Waria* friends and the* Waria* coordinator were reported to be the source of information about the HIV/AIDS-related health services available for* Waria*. Participants reported that* Waria* had a monthly informal meeting through which they were acquainted with each other and shared information about their personal health and health services accessible to them:

“I receive information about HIV counselling, HIV test, ARV, STIs test, viral load test, and CD4 check from my [*Waria*] friends. Initially, I did not want to access, I did not want to do the test [HIV test] but since 2012 I encouraged myself and started attending VCT regularly every three months and in December 2012 I was diagnosed with HIV” (R1: 55 years old).

“I heard about the [HIV/AIDS] services for the first time from the* Waria* coordinator” (R17: 44 years old).

“We get together every month at our houses in turn and we share information about our health, sexually transmitted infections, condoms, health services,  .... “ (R21: 42 years old).

### 3.3. Social Supports from Fellow* Waria*

#### 3.3.1. Emotional Support

Emotional support from* Waria* friends and the* Waria* coordinator that was expressed through kind attention, caring, listening, and encouraging words seemed to play important role in supporting the study participants to seek health services. All the study participants commented that they had positive emotional support and encouragements from their fellow* Waria *and their coordinator which enhanced their HIV/AIDS-related health service accessibility including attending VCT, checking CD4 level and viral load, testing for other STIs, and accessing ARV:

“I was encouraged by my [*Waria*] friends to make use of the HIV/AIDS services available for us [*Waria*] especially VCT. They are aware of how susceptible we are to the infection. Initially, I did not want to do it but a close [*Waria*] friend of mine encouraged me and she said “you are strong, you can do it. Do you want to die like the others?” Finally, I did the test in 2012 and was diagnosed with HIV” (R25: 43 years old).

“I did not want to use the [HIV/AIDS-related health] services because I was scared of the disease. My physical condition was so weak, and I was so skinny. I was encouraged by my friends [*Waria*] to attend VCT, one of them said ‘sister, you have to do the VCT, look at your condition, it is very simple, just do it'.  .... I attended the VCT and the result was that I am infected with HIV. I am grateful that we [*Waria*] have good relationship and continue to encourage each other to do regular medical check-up and access ARV” (R12: 50 years old).

“I feel that I am not alone, there are my other friends [*Waria*] and also mami [the term* Waria* used to address the* Waria* coordinator] who care, support and look after me. We know our health conditions and needs, and are dependent on each other, so why should I be afraid of attending VCT or using health services? The services accessibility opportune to me is also beneficial for my life” (R8: 42 years old).

#### 3.3.2. Informational Support

Information about HIV/AIDS-related health services provided by their fellow* Waria* and the* Waria* coordinator and shared among them personally or in their monthly meetings was also reported as one of the supporting factors for the study participants in the service accessibility. The majority of them expressed that they knew about HIV/AIDS-related health services from the information shared personally or jointly by their fellow* Waria* and the* Waria* coordinator who supported them to make use of the services:

“.... I used to be sick very often and a [*Waria*] friend of mine informed me about the possibility of HIV/AIDS-related service accessibility in Yogyakarta. She asked me to come to Jogja [nick name of Yogyakarta] and do the test [HIV test]. I refused because I did not know anything about HIV infection and HIV test, but after thinking about it for a while I decided to come here and did the test. I came here in 2007 and was diagnosed with HIV” (R2: 51 years old).

“After I was diagnosed with HIV, I felt like I did not want to live anymore. One day, I chatted with mami Rose [pseudonym, the* Waria* coordinator] on Facebook and she told me about the HIV treatment and encouraged me to do it.  .... Other [*Waria*] friends are also very supportive, we are connected to each other and we know each other very well” (R27: 39 years old).

“A friend [*Waria*] of mine who lives here told me the HIV/AIDS health services that I can access here. So, I came here, did the test and I was [HIV] positive. I would not have done the test and accessed the services if she did not tell me.  .... We also share information about health services in our regular meetings,  ....we have good relationship with each other” (R22: 44 years old).

#### 3.3.3. Instrumental Support

Instrumental support from fellow* Waria *seemed to be another important facilitator of the HIV/AIDS-related health service accessibility among the participants. The study uncovered that all the participants received instrumental support from their fellow* Waria* enabling HIV/AIDS-related health service accessibility for them. The supports included collecting ARV for other* Waria* friends from hospitals or community health centres, contacting ambulance in case of emergency need, and accompanying each other to access health services:

“I don't have any difficulties in using health services here. There are too many friends [*Waria*] willing to help. They can help take the medicine or look for Ambulance in case of emergency or accompany me to hospital. We [*Waria*] support each other” (R4: 39 years old).

“.... Initially, I didn't know how to obtain the treatment, I didn't know where to go but the mami [*Waria* coordinator] sent a [*Waria*] friend to pick me up at home and accompanied me to the hospital, and up to now I continue to use the services” (R13: 47).

“Previously I was accompanied by my friend [*Waria*] to pick up ARV or check my CD4 or viral load because I did not know, but now I can reach the service and use it by myself. Sometimes, my [*Waria*] friend collects the ARV from the hospital on my behalf and I go to take it from her house” (R26: 51 years old).

Another important factor that facilitated the HIV/AIDS-related health service accessibility by the participants was the supportive role played by fellow* Waria* when a* Waria* did not have health insurance and lacked the capacity to receive treatment due to poor financial position:

“There are too many* Waria* friends who do not have the Indonesian National Health Insurance or Community Health Insurance because they do not have national identity card or are not the resident of Yogyakarta. This means they have to pay for every health service they access. I accompany them to Gedongtengen community health centre and they can get free access for HIV/AIDS-related health services  ....” (R5: 39 years old).

“It is not difficult utilise health services because there are other friends [*Waria*] who help take care of everything, so the process is easy and quick because they know the procedures, where to go and whom to speak to. Since six months ago I started receiving ARV at Gedongtengen community health centre, a [*Waria*] friend of mine helped me and other [*Waria*] friends because my Community Health Insurance cannot be used anymore at hospital for service accessibility. It is only for the ones who have Yogyakarta identity card [Yogyakarta resident]” (R20: 37 years old).

#### 3.3.4. Appraisal Support

Appraisal support among* Waria* participants seemed to also help them with HIV-related health service accessibility in the study setting. Such support was provided in the form of feedback on their capability to utilise the services (“What you need to do is telling the doctor that you want the treatment (ART), you can do this” (R9: 32 years old)) and overcoming the side effects taking ARVs (“You can handle side effects of the medicine ….” (R21: 42 years old)):

“I was reluctant to utilise the services because I didn't know what to say once I met nurses or doctors in the hospital. But, after a while I had a conversation with a (*Waria*) friend of mine, she said “Just go and tell them (nurses or doctors) about your health condition and that you want to access ARV. This is not difficult, you can do it” (R4: 39 years old).

“After the first-time taking ARV, I stopped accessing the medicine because I could not cope with the effect; every time I finished taking the medicine I felt like I would die. But my friends kept supporting and telling me that I can handle the side effect. Some said ‘You can handle the side effects as we do. What you need to do is making sure that you eat before taking the medicine and try to sleep after taking the medicine” (R28: 46 years old).

### 3.4. Family and Community Social Support

Family members social support also emerged as a theme supportive of participants' HIV care services accessibility. Three participants commented that their family members knew about their HIV status and encouraged and supported them to continue with medication:

“I am open to my family about my HIV status, I have told them everything. My sister asked me to go back to our home village because she said HIV care service is also available there but I don't want to. She always encourages and supports me to undergo medical check-ups and continue take medicine every time we talk” (R11: 33 years old).

“My family are well informed about HIV, so I have told them that I am HIV positive. The reaction of my family especially my mom was very positive. They see HIV/AIDS as a disease, same as diabetes or cough or stroke and they do encourage and support me to adhere to ARV and do medical check-ups regularly” (R18: 33 years old).

However, the majority of the study participants reported to lack the necessary support from family members such as parents, sibling, or significant others:

“I don't receive any support from my brothers and sisters for the treatment of the infection [HIV], I am the one who supports them financially. I hope my little brother prays for me  ....” (R14: 41 years old).

“.... I do receive ARV and do medical check-ups  ....  No one in my family including my parents, sisters and relatives helps me, but I get a lot of support from my friends [*Waria*] to undergo the [HIV] treatment  ....” (R23: 57 years old).

Lack of family social support for the majority of the participants seemed plausible as they hid their HIV status from family members and relatives. Fear of being rejected by family members and relatives, feeling uncomfortable being together with them if their HIV status was known to family members or relatives, and feeling ashamed to explain HIV status to them were the participants' underlying reasons for hiding HIV status from family members and relatives:

“My family members do not know about my [HIV positive] status  ....  I am afraid they will reject me if I am open about status. It will separate us, so I think it is better to be like this,  ....” (R3: 41 years old).

“All my brothers and sisters do not know that I am HIV positive. I don't want to tell them because I don't feel comfortable being with them if they know about my HIV status” (R15: 53 years old).

“I never tell my family up to now [about HIV status], I feel ashamed to explain to them” (R19: 45 years old).

The social support from the general community enabling HIV/AIDS-related service accessibility among the study participants was also lacking. For example, participants commented that they did not receive any support for healthcare related service accessibility from the general community. It would be reasonable to hypothesize that the lack of the general community support could be a result of* Waria's* concealment of their HIV status due to fear of being rejected by the general community members. However, it was also indicated that people from Yogyakarta were nonjudgemental and it was easier for* Waria* to interact with them:

“.... There is no support from the people in the general community where I live in relation to my health issue. People don't know that I am sick [infected with HIV positive].  .... I don't tell them because I think they don't have much information about HIV/AIDS, so they may get scared and avoid or discriminate me” (R6: 50 years old).

“.... I receive no help from them [the general community members] because they don't know about my HIV status. I hide it from them, I am afraid they may stigmatise me.  ....” (R29: 36 years old).

“People here are not judgemental to us [in relation to their status as* Waria*], I have been living here for years, move from one part to another, and I feel accepted in the communities where I live” (R16: 55 years old).

“.... I feel accepted here, I am a* Waria* but they are not judgemental to me. They often invite me and my [*Waria*] friends to handle some works for their affairs such as wedding ceremonies.  .... We easily interact with each other” (R24: 32).

## 4. Discussion

The study aimed to identify facilitators of HIV/AIDS-related health service accessibility among HIV positive* Waria* in Yogyakarta, Indonesia. The study findings indicate that HIV/AIDS-related health services accessibility was possible to all participants at community health centres and hospitals. Consistent with the findings of studies elsewhere [17–20], this study indicates knowledge about the existence of HIV/AIDS and the availability of HIV/AIDS-related health services to be among the facilitators of accessibility to these services. Additionally, self-awareness of the risk of HIV/AIDS and individual's realisation of the seriousness of this disease (which were found to be the case among the study participants) seemed to support the service accessibility among the study participants. These findings are in line with previous studies [5, 41, 42], where self-perceptions of the risk of HIV infection and its impact have encouraged people to take necessary actions for HIV prevention such as using condoms and accessing treatment including the use of ART. These findings also affirm the HBM construct and its application in a recent study with clients of female sex workers in Indonesia [34, 35, 43] suggesting that a health-oriented behaviour performed by an individual is determined by the personal perceptions of susceptibility to a disease or condition and the subsequent impact on individual's health. Their possession of knowledge and information on these matters seemed to be a result of information shared among them personally or jointly in* Waria* informal regular or monthly meetings. The* Waria's* regular meetings align with the Ottawa Charter for Health promotion. This charter emphasises the creation of, amongst others, creative supportive environments for individuals within communities to take care of each other's health and personal development through the provision of health information and education that enable people to learn throughout life and cope with every health condition facing them [44]. However, findings reported elsewhere [42, 45–48] have also shown that knowledge of HIV/AIDS and its related health services is not always translated into the search for health services due to various reasons, including fear of a positive result of HIV testing, stigma, and discrimination from family and community members, physical abuse, and concerns about confidentiality, long distance travel to healthcare facilities, and transportation costs. This indicates that higher knowledge of HIV/AIDS and its related health services is not necessarily associated with the service accessibility [49–51]. This also shows that knowledge alone does not determine motivation to health-seeking behaviour or health service accessibility. Motivation to act or health service accessibility should also be supplemented by both psychological and social components of support. However, in line with the HBM [34, 35, 43], the findings of the current study indicate that the study participants understand the benefits of HIV/AIDS-related services accessibility to their health.

According to the social network framework [30, 31], issues such as frequency of meetings, reciprocity of ties, and intimate relationship among individuals in a social network can provide opportunities for social support (emotional, instrumental, informational, and appraisal support) that can have positive impacts on health through seeking help. The social support among individuals within* Waria* community expressed by the study participants seemed to reflect the strong connections and social relationships among themselves, which in turn enabled them to share information about their personal health, HIV/AIDS, and health services available to access. In conformity with the previous findings and the social network and support frameworks [21, 23, 30, 31], this study suggests that the participants were emotionally supported by their fellow* Waria *and the* Waria* coordinator through kind attention, caring, listening, and encouraging words to further HIV/AIDS-related health service accessibility. Such support seemed to stem from their understanding of their health conditions and needs and interdependence with one another. These findings are in conformity with previous results on the positive effects of health informational support on health service accessibility among different population groups of patients [21, 52, 53]. Besides, it should also be noted that the dissemination of HIV/AIDS-related health information among the study participants as well as* Waria* communities in the study setting was enabled by good social relationships and social networks among them, which is in line with the findings of previous studies by Kim and colleagues [22] and Bliezber [54]. Instrumental supports such as* Waria* helping each other to collect ARV from hospital, contacting ambulance in case of emergency, accompanying each other to utilise health services, and finding solutions for others who did not have capability to pay for health services were additional facilitators to the HIV/AIDS-related service accessibility among* Waria* populations. These findings are consistent with the results of a recent study [24], reporting accompanying of sex partners and friends to HIV/AIDS-related services as a strong supporting factor of the service accessibility among transgender persons and men who have sex with men populations. The act of being accompanied by a friend increases motivation and reduces anxiety related to the HIV/AIDS service accessibility. Appraisal support from fellow* Waria* in the form of feedback on their capability to utilise HIV/AIDS-related health services was another social support reported to increase the service accessibility of the study participants. The positive effects of* Waria *social support reported in this study affirm the significance of the role that social support (including from the family [55, 56] and the broader social network) plays in improving the health outcomes of vulnerable populations in general [57], and not only in the HIV/AIDS-related health service accessibility. On the other hand, however, because of factors that have been discussed elsewhere in this paper such as HIV stigma, some study participants seemed to conceal their HIV status from their potential social networks, including family and community members due to fear of HIV stigma, rejection, and shame, resulting in failing to benefit from these networks. However, it should also be noted that these were perceived stigma and subsequent effects because none of them reported to have experienced any of these. On the contrary, they reported Yogyakarta people to be not judgemental towards their* Waria* status enabling them to meet and discuss health and HIV related issues more easily than they would be if they were elsewhere in the nation.

### 4.1. Study Limitations and Strengths

There are several limitations to note. Given that the participants recruited for study were regular users of HIV/AIDS-related health services and several of them worked as HIV/AIDS volunteers at the two NGOs in the study setting, it seems that they were fully aware of HIV/AIDS and the related health services that were available for them. Besides, the use of snowball sampling technique would have led to under-sampling of* Waria *outside of the social network of the current participants, providing incomplete overview of facilitators of as well as barriers to accessing HIV/AIDS-related services in Yogyakarta. Therefore, the study results may not be transferable to other* Waria* population groups in settings with different conditions. However, to our knowledge, this is the first qualitative inquiry to focus on identifying facilitators of HIV/AIDS-related service accessibility among* Waria *populations in the Indonesian context. The results of this study can be used to partly give information about the development of strategies and interventions to address HIV/AIDS-related healthcare for* Waria* populations in other parts of Indonesia and globally. Future studies on this topic, which cover a larger range of* Waria* populations from different settings, are recommended.

## 5. Conclusions

The current study reports knowledge of HIV/AIDS and its related health services and social support, including emotional, informational, and instrumental support, as the facilitators of the access to HIV/AIDS-related healthcare services among* Waria* populations in Yogyakarta. Good social relationships and social networks among* Waria* people may explain the strong social support they provided for one another regarding the accessibility of HIV/AIDS-related services. As a result, the participants were aware of HIV/AIDS and the available healthcare services and had regularly used the services. In addition, this study also reports the lack of social support from family and community members for the participants to use the services, which might be the consequence of the concealment of HIV status from family and community members by the study participants due to various reasons such as perceived stigma. The findings indicate the need for HIV/AIDS education and HIV/AIDS-related health service information dissemination for* Waria* populations in other settings in the country and globally to increase* Waria*, their family, and community members HIV/AIDS service accessibility and to raise their awareness of HIV/AIDS and acceptance of HIV/AIDS positive people. Furthermore, these findings illustrate the importance of the establishment of* Waria* peer-support groups within* Waria* communities and the involvement of* Waria* in HIV/AIDS activities and programs, which may increase their awareness of HIV/AIDS and of HIV/AIDS-related health service accessibility.

## Data Availability

The data used to support the findings of this study are included within the article.

## Supplementary Materials

Supplementary Materials Table 1: COREQ checklist.
